# The role of miR-24 as a race related genetic factor in prostate cancer

**DOI:** 10.18632/oncotarget.15016

**Published:** 2017-02-02

**Authors:** Yutaka Hashimoto, Marisa Shiina, Taku Kato, Soichiro Yamamura, Yuichiro Tanaka, Shahana Majid, Sharanjot Saini, Varahram Shahryari, Priyanka Kulkarni, Pritha Dasgupta, Yozo Mitsui, Mitsuho Sumida, Guoren Deng, Laura Tabatabai, Deepak Kumar, Rajvir Dahiya

**Affiliations:** ^1^ Department of Urology, VA Medical Center and UCSF, San Francisco, CA 94121, USA; ^2^ Department of Pathology, Veterans Affairs Medical Center and University of California at San Francisco, San Francisco, CA 94121, USA; ^3^ Julius L. Chambers Biomedical/Biotechnology Research Institute (BBRI), Department of Pharmaceutical Sciences, North Carolina Central University, Durham, NC 27707, USA

**Keywords:** race related prostate cancer, miRNA, pathway modulation, DNA methylation

## Abstract

The incidence of prostate cancer (PCa) among African-Americans (AfA) is significantly higher than Caucasian-Americans (CaA) but the genetic basis for this disparity is not known. To address this problem, we analyzed miRNA expression in AfA (*n* = 81) and CaA (*n* = 51) PCa patients. Here, we found that miR-24 is differentially expressed in AfA and CaA PCa patients and attempt to clarify its role in AfA patients. Also, the public sequencing data of the miR-24 promoter confirmed that it was highly methylated and down-regulated in PCa patients. Utilizing a VAMCSF and NDRI patient cohorts, we discovered that miR-24 expression was linked to a racial difference between AfA/CaA PCa patients. Interestingly, miR-24 was restored after treatment of PCa cells with 5Aza-CdR in an AfA cell line (MDA-PCa-2b), while restoration of miR-24 was not observed in CaA cells, DU-145. Ectopic expression of miR-24 showed decreased growth and induced apoptosis, though the effect was less in the CaA cell line compared to the AfA cell line. Finally, we found unique changes in biological pathways and processes associated with miR-24 transfected AfA cells by quantitative PCR-based gene expression array. Evaluation of the altered pathways showed that *AR, IGF1, IGFBP5* and *ETV1* were markedly decreased in the AfA derived cell line compared with CaA cells, and there was a reciprocal regulatory relationship of miR-24/target expression in prostate cancer patients. These results demonstrate that miR-24 may be a central regulator of key events that contribute to race-related tumorigenesis and has potential to be a therapeutic agent for PCa treatment.

## INTRODUCTION

Prostate cancer (PCa) is the most commonly diagnosed type of cancer among men in the US, accounting for 30% of new cancers. While the rate of PCa has declined, African-Americans (AfA) have the world’s highest incidence of PCa with more than two-fold greater mortality compared with Caucasian Americans (CaA) (NIH; SEER Stat Fact Sheets). Overall, AfA PCa patients are earlier age and have higher Gleason scores, PSA levels, and incidence of palpable disease [1, 2]. Multiple factors have been correlated with aggressive prostate tumors. For instance, differential cancer-related gene expression in AfA patients contributes to higher grade tumor [3, 4], and epigenetic mechanisms, such as DNA methylation, effects genes regulation [5]. These reports indicate that AfA patients have hyper-methylation of genes in normal or pre-cancerous prostate tissues that may promote malignancy. Thus hyper-methylation of tumor-suppressor genes may be involved in the racial differences between AfA and CaA patients [6, 7]. However, the relationship between these abnormal methylation patterns and prostate carcinogenesis is still unknown.

MicroRNAs (miRNAs) are small non-coding RNA which consists of 19–23 nucleotides and regulate various genes chiefly through mRNA target degradation and translational repression. It has been reported that more than 60% of human protein-coding are regulated by miRNAs [8]. Numerous published reports show that miRNA expression levels are related to cancer progression and patient prognosis. Recently, DNA methylation of CpG islands in miRNA promoters has been shown to limit miRNA expression in various human cancers, including PCa [9, 10]. miRNA expression profiles show differences between healthy tissues and cancers [11]and also during tumor progression [12]. Nevertheless, miRNA expression as a race-related factor in PCa has not been fully investigated. Hence, a major topic is whether African-American men have a higher overall incidence, earlier age of onset, increased clinically advanced disease and increased bone metastases and mortality from prostate cancer due to differentially expressed miRNAs compared to Caucasians. Among the differentially expressed miRNAs, miR-24 is reported to be a tumor suppressor miRNA in prostate cancer [13–16], however its functional role in PCa comparing AfA and CaA has not been examined.

Here, we have examined whether dysregulated miR-24, may help in understanding the genetic basis for prostate cancer health disparities. In this study, we correlate miR-24 expression in AfA and CaA PCa cohorts and show that miR-24 down-regulation in an AfA PCa cell line has a dramatic effects on several pathways as compared to CaA cell line.

## RESULTS

### Down-regulation of miR-24 is significantly correlated with race in AfA and CaA prostate cancer patients

**Figure 1:**
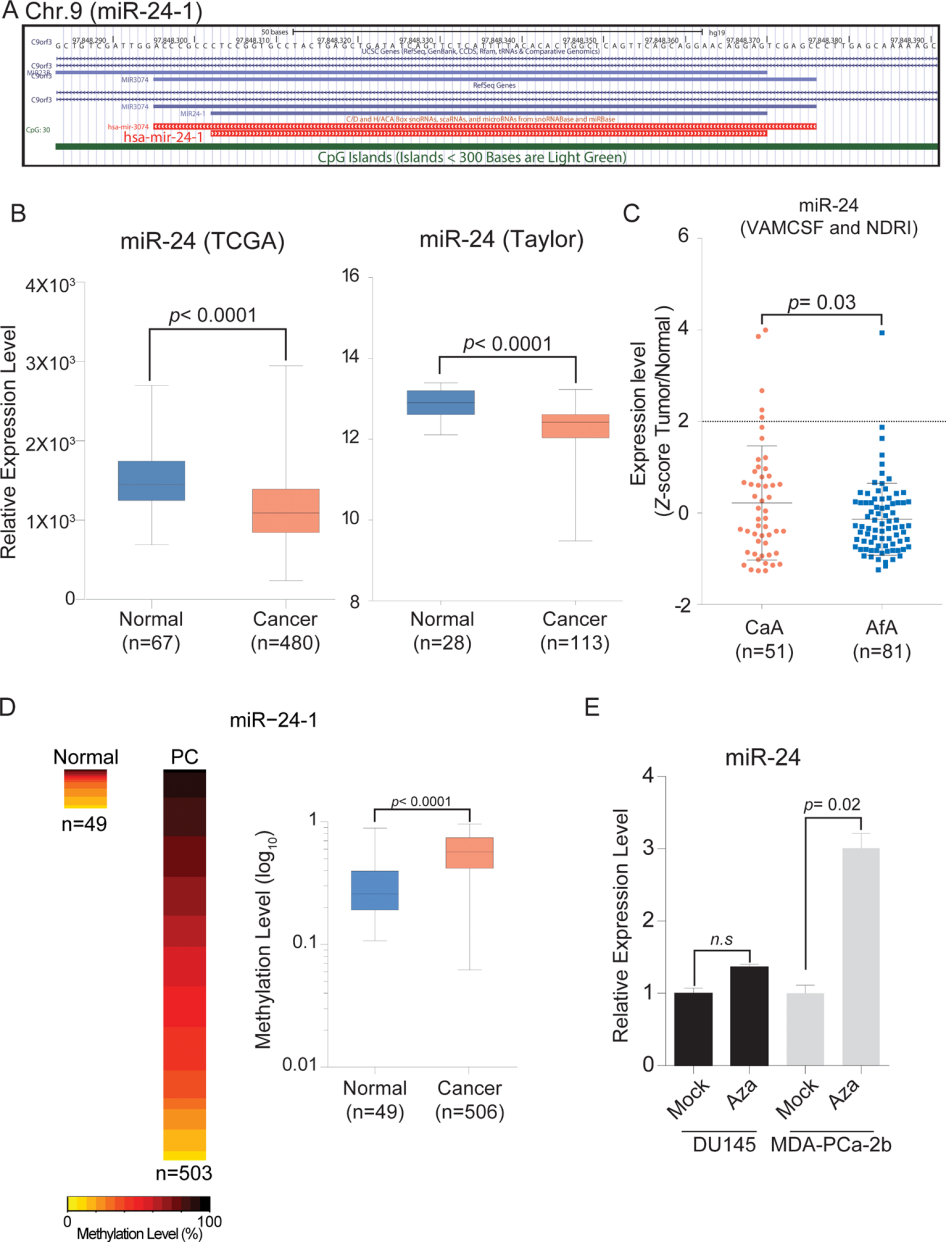
Chromosome location of miR-24-1 and expression levels in PCa tissues (**A**) Schematic diagram of miR-24-1 on chromosome 9 from UCSC genome browser. (**B**) Box plot of miR-24 expression using TCGA (Normal, *n* = 67; PCa, *n* = 480) and GEO (Normal, *n* = 28; PCa, *n* = 113) databases. (**C**) Quantitative PCR expression analysis with AfA and CaA prostate cancer tissues from VAMCSF and NDRI patient cohorts (CaA, *n* = 51; AfA, *n* = 81). Cut off; Z = 2, Fisher’s exact test, *p* = 0.03181, OR = 8.562693, 95% CI [0.9850, 76.77]. (**D**) Heatmap and box plot showing methylation level of TCGA 450K array data (Normal, *n* = 49; PCa, *n* = 506). *P*-value was calculated by Mann-Whitney *U* test. Bar = ± SD. (**E**) miR-24 expression level in 5Aza-CdR treated and untreated PCa cell lines. *P*-value was calculated by two-tailed *t*-test. Bar = ± SEM.

Initially, we confirmed miR-24 expression levels in prostate cancer patients using the The Cancer Genome Atlas (TCGA) and Gene Expression Omnibus (GEO) (GSE21036, Taylor *et al*.) databases. In agreement with previously published reports, miR-24 was significantly down-regulated in PCa samples compared with normal tissues (Figure 1B) in the both of TCGA and Taylor cohorts. However, the TCGA cohort includes very few AfA samples (*n* = 7) and Taylor cohort does not have racial information. We then analyzed a VAMCSF and NDRI cohorts consisting of 81 AfA and 51 CaA samples and found that miR-24 levels were significantly correlated with race using Fisher’s exact test (*p* = 0.0318, OR = 8.562693, 95% CI [0.985, 76.77]) (Figure 1C and Supplementary Table 2).

### 5Aza-CdR restores expression of miR-24 in MDA-PCa-2b

Since total miR-24 expression levels were lower in MDA-PCa-2b cells compared with DU-145 cells (Supplementary Figure 1) and the miR-24-1 promoter was hyper-methylated in PCa patients from the TCGA database (Figure 1A and 1D), we looked to see if reduced miR-24 levels in MDA-PCa-2b cells was due to DNA promoter hyper-methylation. We observed that the expression level of miR-24 was increased after 72 hours treatment with 5Aza-CdR in MDA-PCa-2b but not in DU-145 cells (Figure 1E).

### miR-24 over-expression affected on the cell viability of AfA PC cell line MDA-PCa-2b more than that of CaA cell line DU-145

**Figure 2:**
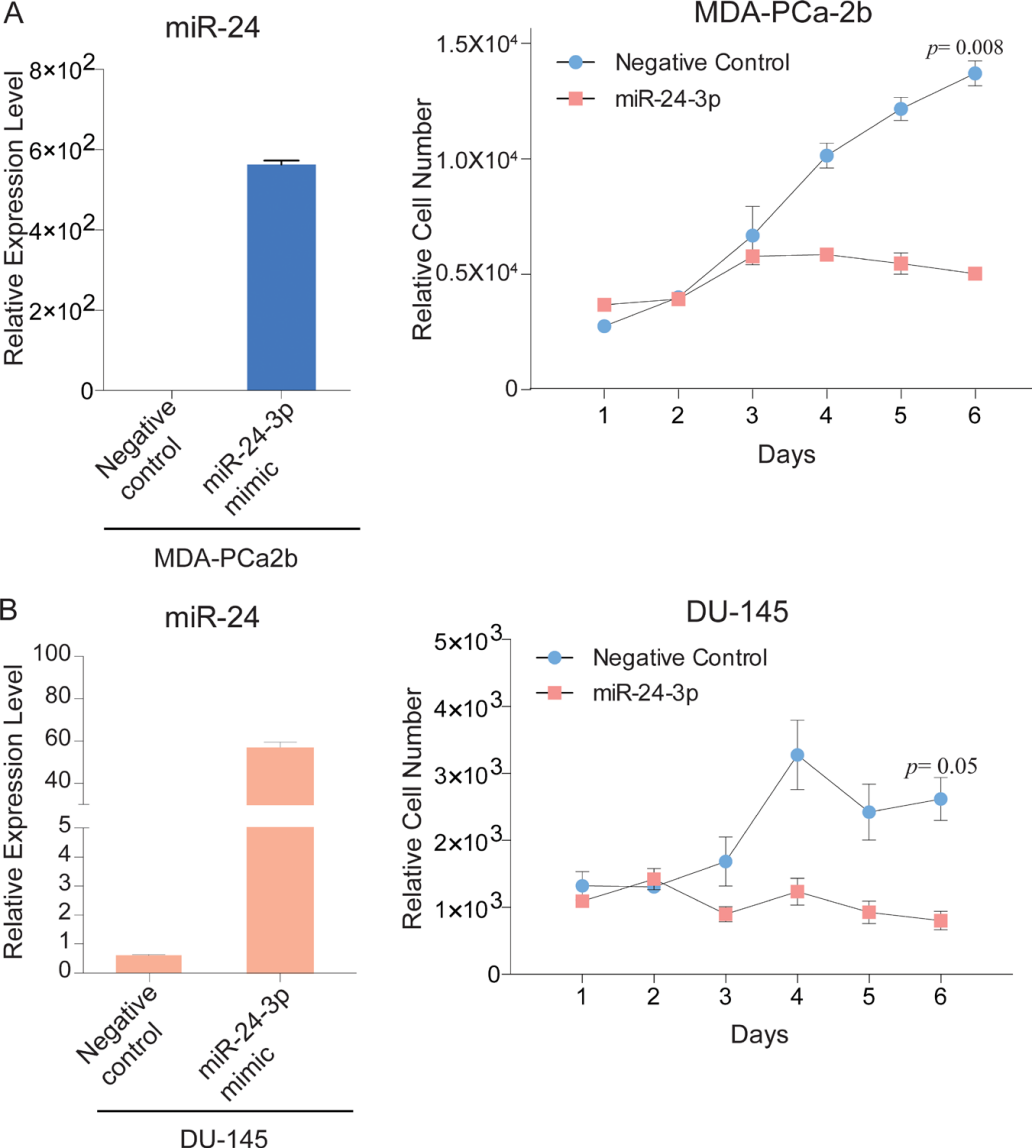
miR-24 over-expression decreases PCa cell proliferation (**A**, **B**) Left bar graphs, Relative expression level of miR-24 after transfection with miR-24 mimic and negative control; Right line plots, Proliferation assay of MDA-PCa2b, DU-145 and cells that were transfected with miR-24 mimic or negative control for 6 days. Bar = ± SEM.

To understand race-related functional differences of miR-24, we performed cell viability assays after transfection with miR-24 mimic. miR-24 over-expression decreased cell proliferation in both MDA-PCa-2b and DU-145 cells compared with negative controls. Importantly, reduced cell viability by miR-24 was more pronounced in MDA-PCa-2b cells (*p* = 0.008) than DU-145 cells (*p* = 0.05) (Figure 2A and 2B).

### Different mechanisms regulate apoptosis in MDA-PCA-2b and DU-145 cell lines

**Figure 3:**
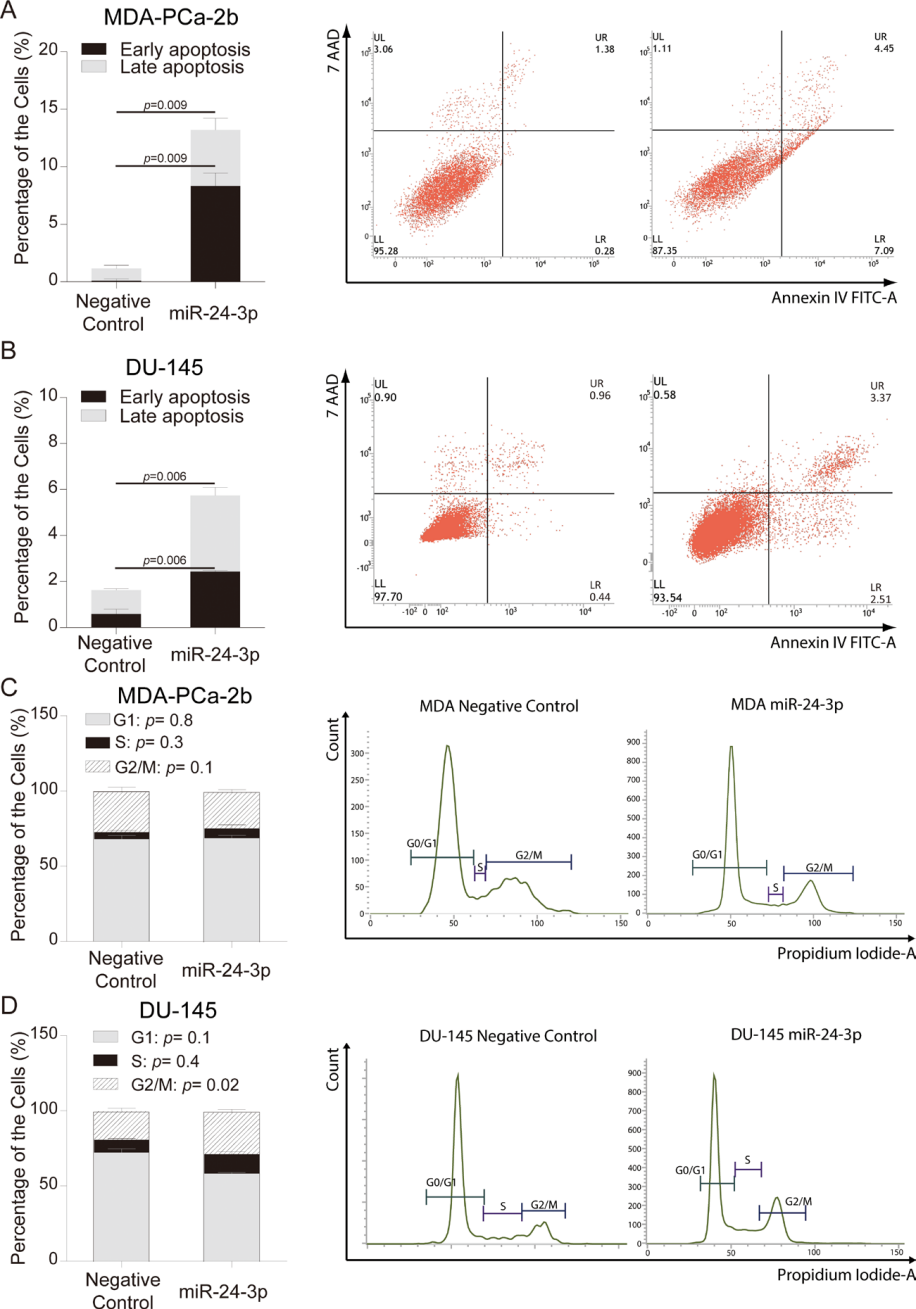
miR-24 over-expression and analyses of apoptosis and cell cycle (**A**, **B**) The percentages of apoptosis in MDA-PCa-2b and DU-145 cells were analyzed by flow cytometry after miR-24 mimic or negative control transfection. Results shown are representative of three independent experiments. Bar = ± SEM. (**C**, **D**) Cell cycle analyses of MDA-PCa-2b and DU-145 cells were carried out by flow cytometry after miR-24 mimic and negative control transfection. Bar = ± SEM.

Apoptosis was quantified using Annexin V staining after transfection of miR-24. miR-24 increased (> 11.3-fold) apoptosis in MDA-PCa-2b cells compared to negative control at day six after transfection, while DU-145 cells showed a 3-fold increase of apoptosis at day four (Figure 3A and 3B). Up-regulation of cleaved Caspase-3 was observed in MDA-PCa-2b cells transfected with miR-24, however down-regulation of cleaved Caspse-3 was observed in DU-145 cells (Supplementary Figure 2). Thus AfA and CaA cells apparently have distinct mechanisms for regulation of apoptosis.

We also checked the cell cycle status of DU-145 and MDA-PCa-2b cell lines after transfection with miR-24. The G2/M phase of the cell cycle was slightly increased in DU-145(Controls 18.3% vs Transfected cells 28.3%, *p* = 0.02), while transfected MDA-PCa-2b cells showed no differences in cell cycle distribution compared to negative control (Figure 3C and 3D).

### miR-24 increases expression tumor suppressor genes

**Figure 4:**
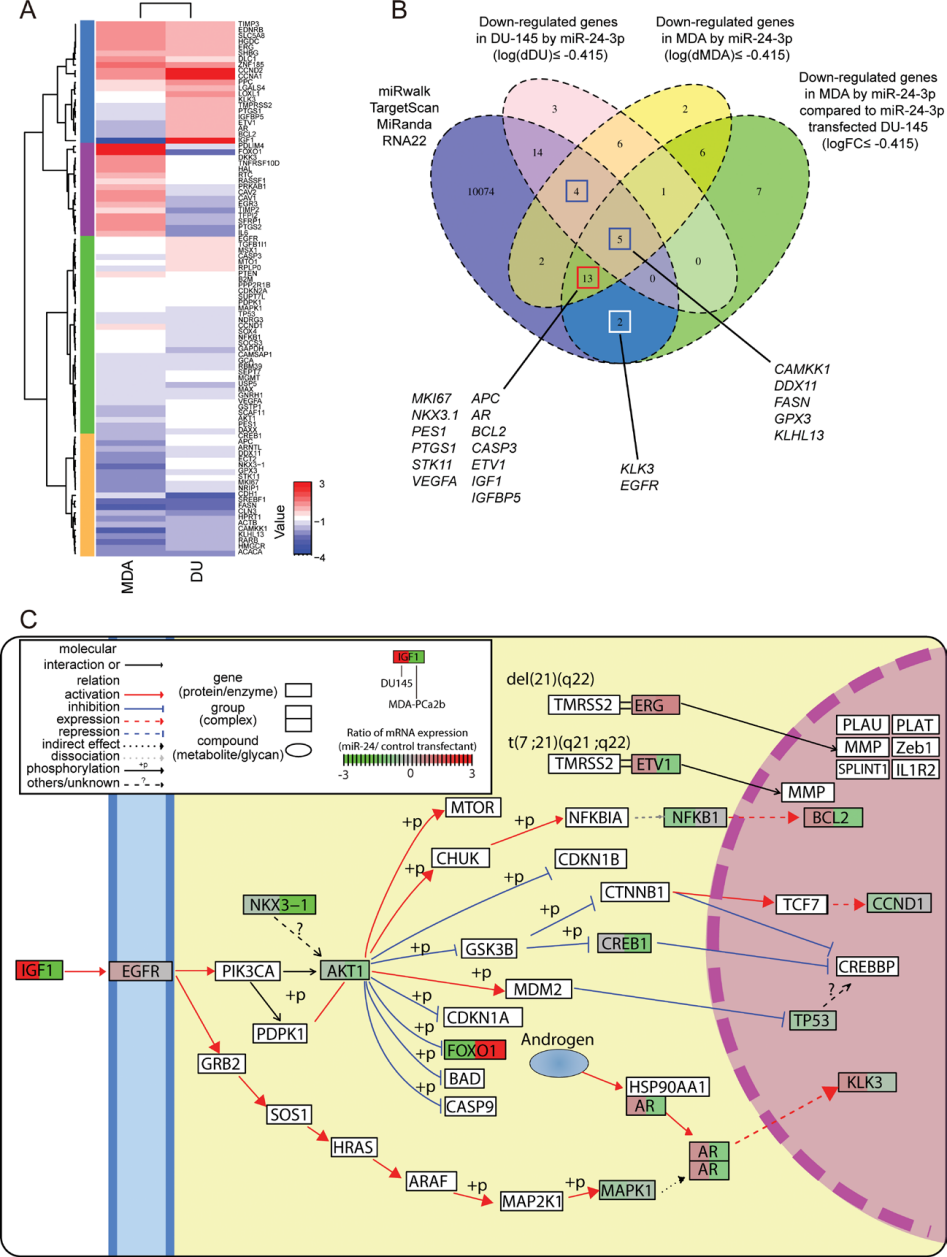
Gene expression profile analyses evaluating the effect of miR-24 on prostate cancer cell lines by qPCR based array analysis (**A**) Hierarchical clustering and heatmap analyses of gene expression profiles in DU-145 and MDA-PCa-2b. The expression values were calculated by dividing values of miR-24 transfectant with that of negative control. (**B**) Venn diagram of miR-24 predicted targets and down-regulated genes after miR-24 transfection in MDA-PCa-2b and DU-145. Blue, circle indicates the group of genes which are predicted by at least one database out of miRWalk, TargetScan, miRanda and RNA22. The pink circle show down-regulated genes (≤ 0.75 FC) in DU-145 cells. Yellow circle shows down-regulated genes (≤ 0.75 FC) in MDA-PCa-2b cells. Green shows genes that are down-regulated only in MDA-PCa-2b (≤ 0.75 FC), but did not in DU-145 cell. (**C**) Pathway map with representative genes altered by miR-24. “Pathview” analysis mapped qPCR array data to “Pathway in Prostate Cancer”, “Transcriptional Misregulation in Cancer” that has been defined by KEGG pathway database and renders pathway graph with the mapped data. The boxes surrounding gene symbols are illustrated by two colors. Left side color of the box shows expression change in DU-145 cells after miR-24 transfection and right side shows that for MDA-PCa-2b cells. The color indicates gene expression level in each cell line.

To determine which biological pathways are involved in race-related PCa, we did RT2 qPCR-based array profiling using an assay with PCa related genes and compared gene expression profiles in the AfA and CaA cell lines. The results showed different expression levels of several genes related to apoptosis (*ETV1, GNRH1, PTEN, TIMP2, IGF1* and *BCL2*) in MDA-PCa-2b and DU-145 cells (Figure 4A and Supplementary Figure 3).

As mentioned in Materials & Methods, we calculated gene expression log fold-changes to help further define race-related PCa pathways. miR-24 decreased all apoptosis (*ETV1*, *IGF1*, *BCL2* and *CASP3*) and cell cycle related genes (*IGF1*, *PTGS1*, *BCL2*, *CASP3* and *APC*) (logFC ≤ –0.415) that were up-regulated in MDA-PCa-2b as compared to DU-145 (log(dMDA/DU) ≥ 0.585) (Supplementary Figure 3). Indeed, our apoptosis analyses with FACS corresponded to the qPCR array results (Figure 3A). On another note, two (*AR* and *IGF1*) out of three genes in the Androgen Receptor (AR) pathway that were up-regulated in MDA-PCa-2b cells (log(dMDA/DU) ≥ 0.585) showed specific down-regulation after miR-24 transfection (logFC ≤ –0.415) (Supplementary Figure 3). Remarkably, expression of 8 out of 18 genes that assigned in hyper-methylated in PCa was recovered especially in MDA-PCa-2b after miR-24 transfection (Supplementary Figure 3). Genes in the Transcription Factor, and PI3K/AKT pathways were also up-regulated in MDA-PCa-2b and decreased by miR-24 over-expression (4 out of 7 and 3 out of 5, respectively) (Supplementary Figure 3), while miR-24 has not decreased these genes in DU-145. Interestingly, Fatty Acid Metabolism pathway related genes were also down-regulated in MDA-PCa-2b after miR-24 transfection. These results show that these three pathways were more activated in MDA-PCa-2b cells compared to DU-145 and may be important for understanding the role of genetic factors in race related prostate cancer (Supplementary Figure 3, Figure 4A and 4C).

### miR-24 targets genes in multiple cancer-related pathways

In order to confirm whether the pathways that we mentioned above are statistically significant, we performed conventional pathway enrichment analysis with ConcensusPathDB (CPDB). Using CPDB analysis also detected that the Prostate Cancer, and IGF Signaling Pathway were suppressed at the mRNA level in miR-24 transfected MDA-PCa-2b cells compared with controls (*p* = 5.87E-11 and *p* = 1.17E-04) (Supplementary Table 3), while there were no reductions in expression in DU-145 cells. In addition to these three pathways, genes that were down-regulated only in MDA-PCa-2b cells by miR-24 transfection, were significantly enriched in the PI3K-Akt and AR Signaling Pathways (*p* = 2.79E-05 and *p* = 8.73E-05) (Supplementary Table 3) in agreement with the results of our calculations.

To evaluate whether miR-24 caused the reduction of these genes directly or indirectly, we collated the down-regulated genes with a miRNA target prediction database “miRWalk”. We looked at the potential targets of miR-24 that were annotated in at least one of the four databases (TargetScan, RNA22, miRanda and Pictar) using miRWalk. In total, 10114 genes were registered in the four databases without overlaps. The nine genes (indicated by blue empty boxes) annotated as miR-24 targets and down-regulated in both DU-145 and MDA-PCa-2b are shown in Figure 4B (log(dDU) ≤ –0.415 and log(dMDA) ≤ –0.415). Five (*CAMKK1*, *DDX11*, *FASN*, *GPX3* and *KLHL*) of those nine genes were decreased substantially in MDA-PCa-2b cells compared to DU-145 cells (logFC ≤ –0.415). We also have identified 13 genes (indicated by red empty boxes) which were repressed in MDA-PCa-2b by miR-24, but not decreased in DU-145 (log(dDU) ≥ –0.415, log(dMDA) ≤ –0.415 and logFC ≤ –0.415). This group included several well-known oncogenic genes such as AR, ETV1, and IGF1. These genes are highlighted in the KEGG pathway map by R package “pathview” (Figure 4C and Supplementary Figure 4). Two genes shown in white boxes are also important as PCa biomarkers and in PCa cancer development. However down-regulation of *KLK3* and *EGFR* in MDA-PCa-2b were lesser than cut-off value (log(dMDA) ≥ –0.415) (Supplementary Figure 3).

### miR-24 down-regulates the PCa related oncogenes

**Figure 5:**
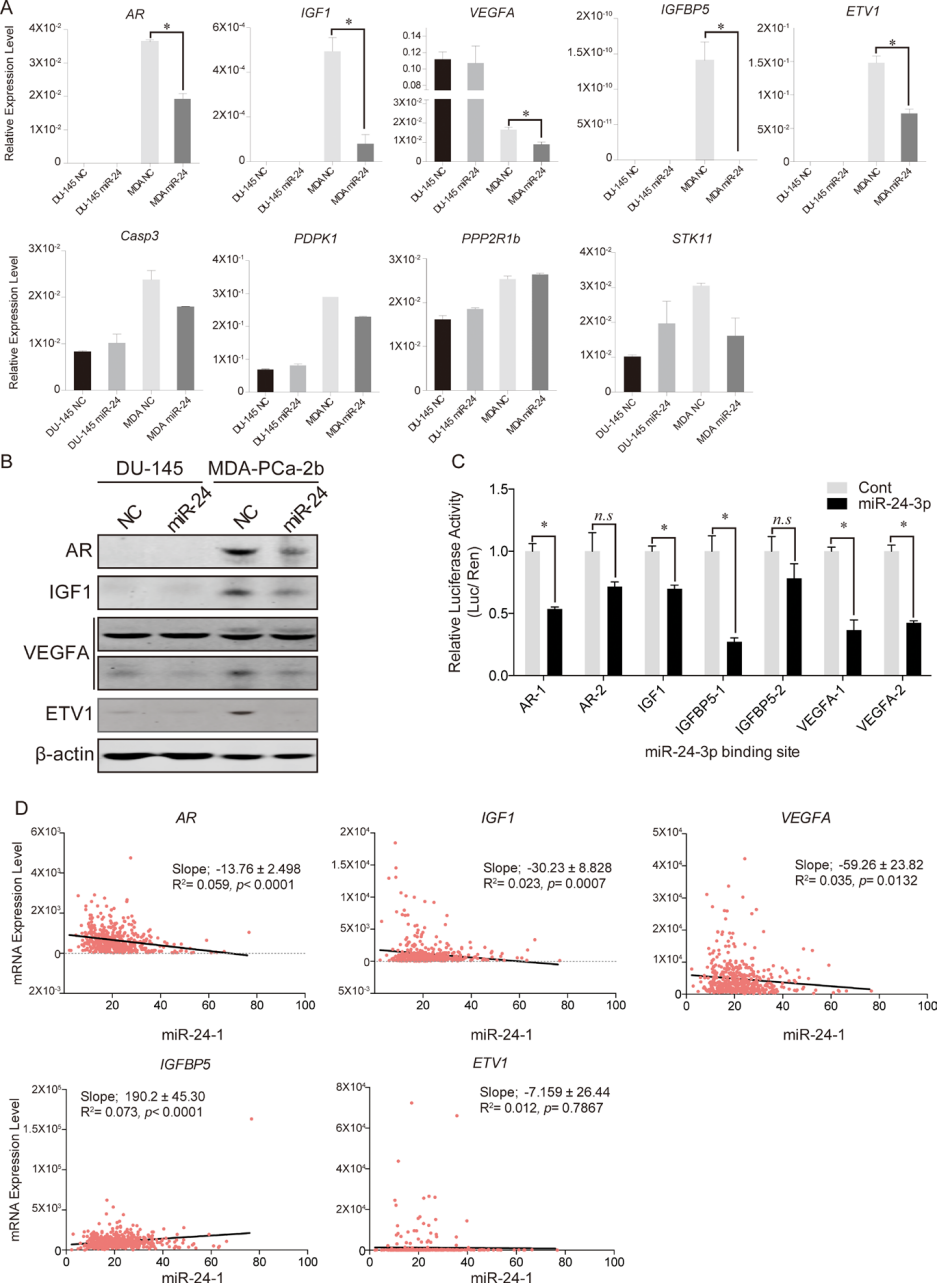
Predicted target gene expression changes after miR-24 induction (**A**) qPCR analyses for the predicted target genes of miR-24. We checked candidate gene expression levels after miR-24 over-expression. *p < 0.05, Bar = ± SEM. (**B**) Western blotting of miR-24 target genes. (**C**)The results of Dual Luciferase Reporter Assays. Except for IGF1, all genes have two different binding sites (for instance; AR-1 and AR-2 mean that miR-24 was predicted that it may bind to two different site of AR mRNA). *p < 0.05, n.s; no significance, Bar = ± SEM. (**D**) Linear regression model of mRNA expression levels between miR-24-1 and its five target genes in human PCa samples.

We then confirmed our qPCR array results by quantitative PCR. Eight genes were selected from the 13 genes which were particularly down-regulated in MDA-PCa-2b cells and *PPP2R1b* was used as a negative control. Significant down-regulation of 5 genes was confirmed by using qPCR. Of note, *AR*, *IGF1*, *IGFBP5* and *ETV1* expression were not been detected in DU-145 cells, while expression of both genes was dramatically reduced by miR-24 in MDA-PCa-2b cells (Figure 5A). The decreased protein expression levels of AR, IGF1, ETV1, and one of VEGFA splicing variants (lower column) were observed in MDA-PCa-2b after miR-24 over-expression (Figure 5B). To determine whether the predicted miR-24 binding sites of target genes would function for miRNA binding, we carried out Dual Luciferase Reporter Assay. We referred the miR-24 binding sequences by TargetScan and RNA22 (Supplementary Table 1) and carried out Dual luciferase reporter assay with the vectors containing miR-24 binding site. Two days after co-transfection each vector and miR-24, the relative luciferase activities of AR, IGF1 IGFBP5, and VEGFA binding site coding vectors significantly reduced compared to negative control transfected cells. However, other binding sites of AR and IGFBP5 were not significantly changed (Figure 5C).

In silico analysis with TCGA database indicated inverse relationships between miR-24-1 and target gene expressions (AR, IGF1, and VEGFA) in PCa tissues, respectively (Figure 5D).

### AR expression levels reciprocally associated with miR-24 expression in AfA PCa tissues

**Figure 6:**
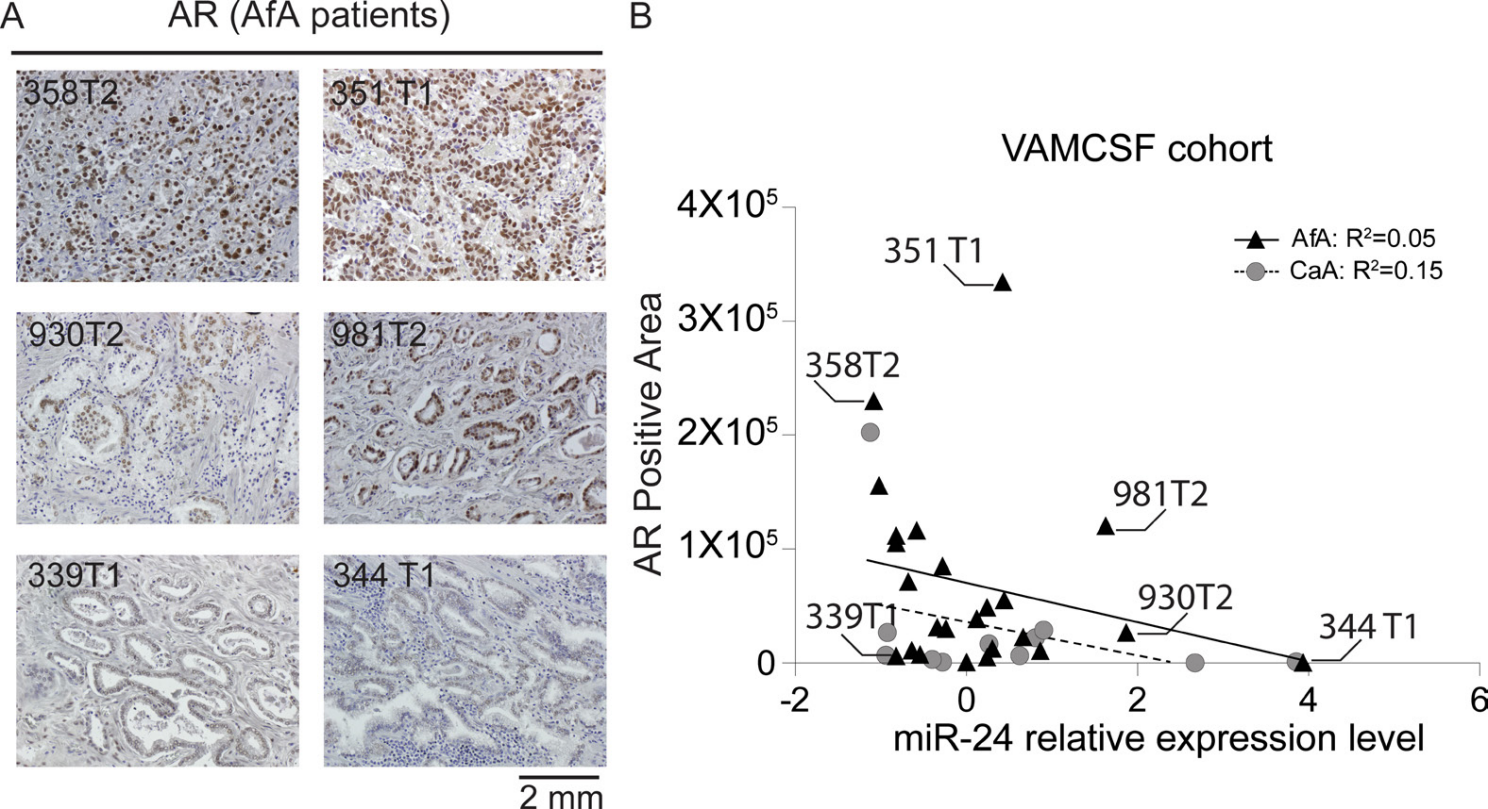
Analyses of AR protein expression in AfA PCa tissues (**A**) Immunohistochemical staining of AR protein expression. The staining is representative of 24 AfA (Black-filled triangles) and 11 CaA (Grey-filled circles) PCa tissues. The number shown in the IHC image indicates the sample ID that is the same as shown in Figure 6B. (**B**) Association of AR expression positive area and miR-24 expression level in AfA PCa and 11 CaA tissues.

To confirm whether miR-24 expression impact on AR expression level in AfA PCa patients. We performed immunochemical staining for 24 AfA (Figure 6A) and 11 CaA PCa tissue slides and compared AR and miR-24 relative expression levels by linear regression model (Figure 6B). The AR positive area in the PCa tissues shows reciprocally associated with miR-24 expression level in AfA PCa patients of VAMCSF cohort as well as CaA samples (Figure 6B). However, The AR positive area was larger in AfA samples compared to the CaA derived samples (Mann-whitney U test; *p* = 0.03).

## DISCUSSION

A number of studies have shown differential gene expression patterns in AfA and CaA PCa (18–22). These reports suggest that those may be race-related genetic factors in PCa. However to date, identification of these genes has not been accomplished. We hypothesized that miRNAs may be key factors in abnormal gene regulation in AfA PCa patients. In this study, we have shown that miR-24 is a potent tumor suppressor in a AfA PCa cell line.

Previous studies of kidney cancer [23], gastrointestinal cancer [24], esophageal and bladder cancer [25, 26] including PCa [27] have shown that miRNA levels are decreased in tumors due to DNA promoter hyper-methylation. Thus, we also analyzed the methylation status of miR-24-1 and -2. miR-24-1/ -2 are located on chromosomes 9 and 19, respectively. In silico analysis in TCGA database showed hyper-methylation of miR-24-1 in PCa patients. Thus, we considered the possibility that miR-24 expression was decreased in AfA prostate cancer patients due to promoter hyper-methylation. In MDA-PCa-2b cell lines, 5Aza-CdR treatment restored miR-24 expression but not in DU-145 cells. This result further supported the idea that hyper-methylation of CpG islands may suppress miR-24-1 expression thereby generating a difference in miR-24 expression between the two races.

In this study, we identified several biological pathways that are related to prostate cancer that showed different alterations in the AfA cell line compared with the CaA derived cell line by CPDB analysis (Supplementary Table 3). These pathways are well-known as critical in the development or suppression of prostate cancer. miR-24 is involved in the regulation of these pathways, prohibiting cancer development in AfA cell lines.

According to published reports, some AfA PCa patients have higher serum PSA (tumor volume per unit of PSA adjusted for prostate weight) than CaA patients. Tangen *et al*. have reported that PSA-based screening for PCa could have benefit on the overall survival of AfA PCa patients with hormone sensitive and metastatic prostate cancer [28, 29]. Identification of AR pathway suppression by miR-24 (Figure 4C) may explain why AfA prostate cancer patients had higher PSA levels. Similarly, IGF1 and Akt signaling pathways were significantly reduced in the AfA derived cell line compared with the CaA cell line. Indeed, both pathways have been reported to be involved in AR regulation and established pathways that promote human prostate cancer [30–33] (Figure 4C). Besides a change in AR expression, the IGF signaling affects PI3/Akt pathway and activations of these two signaling pathways could contribute to PSA up-regulation in AfA patients. We have already reported that the activated Akt pathway and miR-34b dysregulation induces prostate cancer development and poor prognosis [9]. In AfA patients, dysregulations of miRNA mediated gene regulation may result in malignant PCa.

We detected restorations of tumor suppressor genes by miR-24. As analyzed by Western Blotting, miR-24 decreased EZH2 and this alteration may cause histone methylation changes and restorations of hyper-methylated genes in cancer. In several PCa cell lines, EZH2 affects SFRP1 and IGFBP7 expression levels by histone and DNA methylation [34]. Further studies are needed to document the epigenetic changes induced by miR-24.

The race-related responses to the ectopic induction of miR-24 between AfA and CaA have not been reported previously. Therefore, we performed quantitative PCR-based expression array analysis using MDA-PCa-2b and DU-145 cell lines. Some, 38.3% and 45% of the genes that were annotated in the array were down-regulated after ectopic expression of miR-24 in DU-145 and MDA-PCa-2b. Among them, about 50 % of the genes decreased by miR-24 transfection were annotated by miRWalk. These alterations prompted us to focus on conventional PCa related pathways that are regulated by miR-24 rather than individual genes.

miR-24 had a greater effect on cell proliferation and apoptosis in the AfA derived cell line compared to CaA cells. These differences may be attributed to the number of genes which were decreased by miR-24. Following our qPCR-based array, 39 genes identified as prostate cancer related were totally down-regulated in MDA-PCa-2b (Figure 4B and Supplementary Table 3). Furthermore, 23 genes were decreased in the AfA cell line including *AR*, *IGF1*, *IGFBP1*, *ETV1* and *VEGF* which are predicted as miR-24 oncogene targets in the database. In contrast, only 17 genes were specifically decreased in DU-145 cells and 13 of these 17 genes were identified as miR-24 targets in the database. Interestingly, *IGF1*, *ETV1*, *IGFBP5* and *AR* expression levels were significantly lower in DU-145 compared with MDA-PCa-2b. Thus, miR-24 decreased expression levels of these genes only in MDA-PCa-2b cells. Also, *AR* and *IGF1* have been reported to be genetically aberrant in AfA PCa patients and are possibly race-related genetic factors in PCa (35,36). In our results of IHC analyses, AR expression levels were significantly increased in AfA compared to CaA PCa patients (*p* = 0.03) and lower miR-24 expression levels were observed in the samples that showed higher AR expression (Figure 6A, 6B and Supplementary Figure 5). Hence it is possible that abnormal over-expression of race-related PCa oncogenes such as *AR* and *IGF1* in AfA cells is accelerated by down-regulation of miR-24. This result also indicates that the differences in basal AR and miR-24 expression levels in prostate cancer could be one of the reasons for racial disparity between AfA and CaA patients might rise. In this study, we chose AR-negative cell line, DU-145 and AR-positive cell line, MDA-PCA-2b. The comparison with these CaA and AfA cell line by qPCR array would be justified because AR expression levels in CaA were significantly lower than in AfA tissues. On the other hand, *VEGFA* was expressed in both CaA and AfA cell lines. Nevertheless, down-regulation of *VEGFA* expression was observed only in MDA-PCa-2b. Additional studies will be required to explain this difference.

Although 60% of human genes were predicted as target of miRNAs [8], miR-24 was also confirmed as a regulator of various gene in PCa. In addition to the reductions of target genes by miR-24 transfection, our linear regression analysis with TCGA database indicates inverse correlation between miR-24 and each three target genes (*AR*, *IGF1* and *VEGFA*), and supports that miR-24 may directly control these three oncogenes in human PCa (Figure 5B, 5C and 5D).

In summary, this work has shown that the down-regulation of miR-24 correlated with race difference. Also the dysregulation of tumor suppressor miR-24 results in low levels in an AfA derived cell line (MDA-PCa-2b), compared to a CaA cell line (DU-145). Due to the fact that a large number of cancer-related pathways were suppressed after miR-24 over-expression, miR-24 may be a central regulator of key events that contribute to race-related tumorigenesis. Thus miR-24 may be a potential therapeutic candidate for PCa treatment. These results also provide further insight into the molecular mechanisms associated with aggressive PCa in AfA patients.

## MATERIALS AND METHODS

### Cell lines

Human Prostate cell lines MDA-PCa-2b, DU-145 were obtained from the American Type Culture Collection (ATCC). Caucasian American (CaA) derived cell line, DU-145 was maintained in RPMI-1640 medium and HPC1 medium was used for the African American (AfA) derived cell line, MDA-PCa-2b. RPMI-1640 and HPC1 media were supplemented with 10% and 20% fetal bovine serum, respectively with 100 IU/mL penicillin and 100 μg/mL streptomycin. Cells were incubated at 37°C in humidified 5% CO_2_. To facilitate MDA-PCa-2b attachment, Poly-L-Lysine (Sigma–Aldrich) Cell Attachment Protocol was implemented for all cell culture plates and dishes. These human-derived cell lines were authenticated by DNA short-tandem repeat analysis by the ATCC.

### Cell cycle analysis

Cells were harvested, washed and fixed in cold 70% ethanol overnight at –20°C. Cell pellets were stained with PI/RNase Staining Buffer (BD Pharmingen) and incubated for 15 minutes at room temperature in the dark. Cells were analyzed for DNA content by gate excluding doublet cells on BD FACSVerse (BD Pharmingen). All analyses were performed in triplicate and 10,000 gated events/sample were counted.

### Apoptosis assay

Cells were transfected with miRNAs or negative control and harvested at different time points. Cells were washed in cold PBS, resuspended in 1x binding buffer and stained with Annexin V-FITC and 7AAD viability dye (Annexin V-FITC/7AAD kit, Beckman Coulter). After 15 minutes incubation at room temperature in the dark, cells were washed and analyzed using BD FACSVerse (BD Pharmingen).

### Western blot analysis

Cells were with NP-40 (Thermo Scientific) plus Halt Protease and Phosphatase Inhibitor Cocktail (Thermo Scientific). Protein concentration was measured using the BCA Protein Assay (Thermo). Western blots were performed using NuPAGE 4–12% Bis-Tris Protein Gels (Invitrogen). For gel electrophoresis, the iBlot 2 Dry Blotting System (Invitrogen) was used with MES buffer (Invitrogen) and transferred onto nitrocellulose transfer membrane. Membranes were incubated with Odyssey blocking buffer (Li-Cor) prior to incubation with primary antibodies overnight at 4°C. Goat anti-rabbit IgG (H+L) 800 CW or goat anti-mouse (H+L) 680RD was applied for 45 minutes at room temperature (1:15000, LI-COR) before washing with PBS with Tween 20. An Odyssey Infrared Imaging System Scanner was used to generate immunoblot images and the LI-COR Odyssey scanner and software (LI-COR Biosciences) were utilized for band quantification. The antibodies used were specific for AR (5153; Cell Signaling; 1:2000), ETV1 (PA5-41484; ThermoFisher Scientific; 1:1000), IGF1 (PA5-27207; ThermoFisher Scientific; 1:1000), VEGF (PA5-16754; ThermoFisher Scientific; 1:200), and β-actin (3700; Cell Signaling; 1:2000).

### Measurement of cell viability

Cell proliferation was estimated by Victor X2 (PerkinElmer) and Cell Titer Glo Luminescent Cell Viability Assays (Promega) were performed every 24 hours for 6 days after transfection of the miR-24 mimics (Thermo Fisher Scientific) following the manufacturer’s instructions. mirVana miRNA Mimic Negative Control #1 was transfected as a control (Thermo Fisher Scientific).

### 5Aza-CdR treatment of cells and RNA extraction

For demethylation study cells were treated daily with 10 μmol/L 5-Aza-Deoxycitydine(5Aza-CdR) (Sigma–Aldrich) for 72 hours. Total RNA was isolated using a miRNeasy mini kit (Qiagen).

### In silico DNA methylation analysis

DNA hypermethylation of the miR-24-1 promoter region, was validated using TCGA data portal (https://tcga-data.nci.nih.gov/tcga/). The analyzed samples included 49 normal and 506 prostate cancer samples. We obtained TCGA DNA methylation data from the HumanMethylation 450 BeadChip (level 3).

### Quantitative real-time reverse transcription–polymerase chain reaction

Real-time reverse transcription–polymerase chain reaction (RT–PCR) was carried out using a Quant Studio 7 PCR System, TaqMan Universal PCR Master Mix, TaqMan Reverse Transcription kit and TaqMan miRNA assays (Thermo Fisher Scientific) according to the manufacturer’s instructions. The expression levels of miRNA were determined on the amount of target miRNA relative to that of RNU48 as a control to normalize the initial input of total RNA. A QuantiFast SYBR Green PCR Kit was also utilized for gene expression analysis of miR-24 targets. The primers used for SYBR Green-based qPCR analyses are listed in Supplementary Table 1.

### Transfection with miRNA mimic

In order to induce miR-24 expression, cells were transfected with mirVana miRNA mimics (100nM) (Thermo Fisher Scientific) using Lipofectamine RNAi Max (Thermo Fisher Scientific). To verify the transfection effect of miRNA mimics, mirVana miRNA Mimic Negative Control #1 (100 nM) (Thermo Fisher Scientific) was included in each transfection experiment.

### RT^2^ profiler PCR array analysis

Expression profiling of 84 genes involved in prostate cancer was performed utilizing human RT^2^ Profiler PCR Array PAHS-135Z (Qiagen) based on SYBR-Green real-time PCR. Three biological replicates were prepared for each miRNA and control transfections and the isolated RNA samples from PC cell lines treated the same condition were pooled. cDNA was synthesized from 2866 ng and 855 ng RNA per pool isolated from MDA-PCa-2b and DU-145 respectively, using RT^2^ First Strand Kit (Qiagen) following the manufacturer’s instructions. SYBR-Green real-time PCR was performed and fold-change calculations were done using RT^2^ Profiler PCR Array Data Analysis.

### Pathway analysis

All expression data obtained from the results of the RT^2^ Profiler PCR Array data was calculated as the logarithm of each gene expression value and processed by global median centering normalization. For pathway mapping, the R package “pathview” was utilized [17]. The pathway analysis was performed by Concensus PathDB (http://consensuspathdb.org).

### Identifying AfA specific oncogenic targets and pathways

To define AfA specific oncogenic targets and pathways of miR-24, we categorized genes according to gene function and calculated log fold-change. We first divided gene expression values of Control MDA-PCa-2b with those of control DU-145 and calculated the logarithm, “log(dMDA/DU)” to compare the basal gene expression levels. Then we calculated gene expression changes after miR-24 transfection in both cell lines by dividing gene expression values for miR-24 transfected cells with expression values of control transfectants took the logarithm and named them “log(dDU)” and “log(dMDA)”, respectively. Finally we obtained “logFC” by subtracting “log(dMDA)” from “log(dDU)”. The lower “logFC” means that the gene was less expressed in MDA-PCa-2b cells than DU-145 cells after miR-24 transfection.

### Dual luciferase reporter assay

The Luciferase reporter vectors were constructed by ligation with the annealed custom oligonucleotides containing the putative target binding sites of 3′-UTR into pmiR-GLO reporter vector (Promega, Madison, WI) (Supplementary Table 1). miR-24 transfected in HEK293T9 cells was co-transfected with 1 ng of the pmiR-GLO vector with the 3′-UTR sequences and luciferase activity was measured 48 hr after transfection using a Dual-Luciferase Reporter Assay System (Promega). Relative luciferase activity was calculated by normalizing to the renilla luminescence.

### Clinical samples

Clinical FFPE (Formaldehyde Fixed Paraffin Embedded) samples of AfA (*n* = 81) and CaA (*n* = 51) and clinical information were obtained from the Urology Tissue Bank of the Veteran Affair Medical Center at San Francisco (VAMCSF; 40 AfA and 51 CaA FFPE samples) and National Disease Research Interchange (NDRI; 41 AfA FFPE samples). The miRNeasy FFPE (Qiagen, Germany) was employed for isolation of RNA and cDNA was synthesized with a TaqMan MicroRNA Reverse Transcription Kit (ThermoFisher Scientific, MA) and checked by Taq-Man miRNA assays with Quant Studio 7. The expression levels of miR-24 in PCa samples were compared with paired normal and normalized by *Z*-score. Statistical analysis for race-relationships between AfA and CaA were performed using Fisher’s exact test.

### Immunohistochemistry

Immunohistochemical (IHC) staining was performed in 24 African-American and 11 Caucasian-American prostate cancer specimens. Vision^™^ UltraVision^™^ Detection System (Thermo Scientific) was used to stain AR proteins. AR (Cell Signaling, 5153) diluted 1:400 in 5% normal goat serum in PBST. DAB substrate was incubate for 10 min. ImageJ calculated AR positive area. Threshold was set by 140.

## SUPPLEMENTARY MATERIALS FIGURES AND TABLES


