# Supplementary material for: The role of miR-24 as a race related genetic factor in prostate cancer
Source: Oncotarget. 2017 Feb 2;8(10):16581–93. doi: 10.18632/oncotarget.15016 (PMC5369986; doi:10.18632/oncotarget.15016)
Supplement: Supplementary file 1 [file oncotarget-08-16581-s001.pdf]

The role of miR-24 as a race related genetic factor in prostate cancer

Supplementary Materials

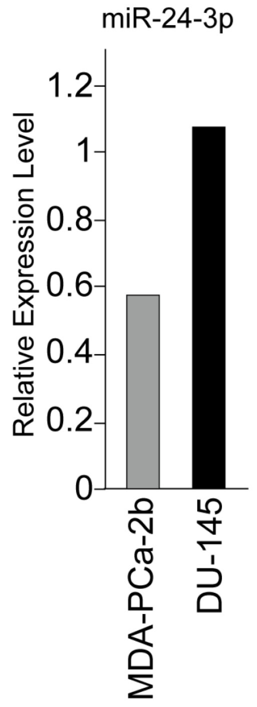

Supplementary Figure 1: miR-24 expression in MDA-PCa-2b and DU-145 cells. Bars indicate miR-24 relative expression level.

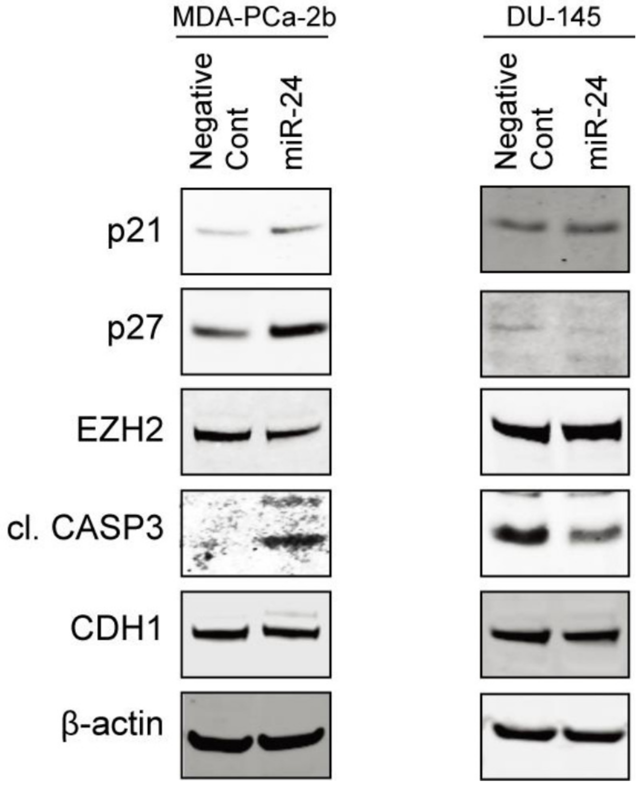

Supplementary Figure 2: Protein expression levels of cell proliferation and cancer related genes. Results of Western blotting after miR-24 mimic or negative control transfection.

Androgen Signaling

Control

Symbol	logFC
AR	11.59
IGF1	7.33
SHBG	0.78
PTEN	0.57
TIMP3	0.00
CCND1	-0.47
NFKB1	-0.93
GNRH1	-1.07
DAXX	-1.27
VEGFA	-1.60
NRIP1	-1.78
EGFR	-2.21
TGFB1I1	-3.50
FOXO1	-6.92
TIMP2	-8.89
IL6	-11.32
CAV1	-13.64

miR-24-3p

Symbol	DU-145	MDA-PCa2b	logFC
IGF1	2.78	-3.07	-5.85
AR	0.72	-0.84	-1.55
NRIP1	-0.36	-1.37	-1.01
VEGFA	-0.13	-0.68	-0.55
EGFR	0.40	-0.03	-0.43
DAXX	-0.38	-0.78	-0.40
TGFB1I1	0.24	-0.04	-0.27
GNRH1	-0.49	-0.68	-0.19
SHBG	0.78	0.79	0.01
PTEN	-0.06	0.09	0.15
TIMP3	0.72	0.89	0.17
CCND1	-0.42	0.07	0.49
NFKB1	-0.67	0.03	0.70
CAV1	-0.73	0.89	1.63
TIMP2	-1.50	0.32	1.82
IL6	-1.34	0.75	2.09
FOXO1	-1.58	2.43	4.01

Apoptosis

Control

Symbol	Normalized
ETV1	13.80
IGF1	7.33
BCL2	3.10
CASP3	1.53
PTEN	0.57
TIMP3	0.00
MAPK1	-0.66
NFKB1	-0.93
GNRH1	-1.07
TP53	-1.52
VEGFA	-1.60
EGFR	-2.21
TIMP2	-8.89
IL6	-11.32

miR-24-3p

Symbol	DU-145	MDA-PCa2b	logFC
IGF1	2.78	-3.07	-5.85
ETV1	0.72	-0.81	-1.53
BCL2	0.65	-0.86	-1.50
CASP3	0.28	-0.57	-0.85
VEGFA	-0.13	-0.68	-0.55
EGFR	0.40	-0.03	-0.43
GNRH1	-0.49	-0.68	-0.19
PTEN	-0.06	0.09	0.15
TIMP3	0.72	0.89	0.17
TP53	-0.59	-0.39	0.19
MAPK1	-0.53	-0.27	0.26
NFKB1	-0.67	0.03	0.70
TIMP2	-1.50	0.32	1.82
IL6	-1.34	0.75	2.09

Cell Cycle

Control

Symbol	Normalized
IGF1	7.33
PTGS1	3.66
BCL2	3.10
CASP3	1.53
APC	0.95
PTEN	0.57
PPP2R1B	0.44
CCND2	0.00
CCND1	-0.47
TP53	-1.52
EGFR	-2.21
CCNA1	-6.60
CAV2	-12.64

miR-24-3p

Symbol	DU-145	MDA-PCa2b	logFC
IGF1	2.78	-3.07	-5.85
CCND2	2.58	0.89	-1.69
CCNA1	2.43	0.89	-1.54
BCL2	0.65	-0.86	-1.50
PTGS1	0.72	-0.56	-1.28
APC	-0.07	-1.34	-1.27
CASP3	0.28	-0.57	-0.85
EGFR	0.40	-0.03	-0.43
PPP2R1B	-0.14	-0.07	0.06
PTEN	-0.06	0.09	0.15
TP53	-0.59	-0.39	0.19
CCND1	-0.42	0.07	0.49
CAV2	-0.50	0.89	1.39

Downregulation in Cancer

Control

Symbol	Normalized
IGF1	7.33
LOXL1	3.53
LGALS4	2.85
USP5	1.00
PPP2R1B	0.44
CLN3	0.22
CCND2	0.00
SLC5A8	0.00
GCA	-0.12
SFRP1	-7.03
TFPI2	-12.06

miR-24-3p

Symbol	DU-145	MDA-PCa2b	logFC
IGF1	2.78	-3.07	-5.85
CCND2	2.58	0.89	-1.69
LOXL1	1.12	-0.18	-1.30
LGALS4	0.71	0.15	-0.56
GCA	-0.38	-0.56	-0.18
PPP2R1B	-0.14	-0.07	0.06
SLC5A8	0.72	0.89	0.17
USP5	-0.83	-0.63	0.20
CLN3	-1.19	-0.85	0.33
TFPI2	-0.96	0.89	1.86
SFRP1	-1.05	0.89	1.95

Fatty Acid Metabolism

Control

Symbol	Normalized
IGF1	7.33
FASN	2.96
CAMKK1	2.41
ACACA	2.10
HMGCR	1.59
SREBF1	1.39
STK11	-0.56
PRKAB1	-2.05

miR-24-3p

Symbol	DU-145	MDA-PCa2b	logFC
IGF1	2.78	-3.07	-5.85
CAMKK1	-0.91	-1.95	-1.05
STK11	-0.32	-1.32	-1.00
FASN	-1.58	-2.23	-0.64
HMGCR	-1.09	-1.40	-0.31
ACACA	-1.25	-1.42	-0.16
SREBF1	-1.66	-1.68	-0.02
PRKAB1	-0.36	0.52	0.88

Metastatic Potential

Control

Symbol	Normalized
KLHL13	1.64
NDRG3	0.69
MAX	0.44
CREB1	0.23
PES1	-0.55
SEPT7	-0.70

miR-24-3p

Symbol	DU-145	MDA-PCa2b	logFC
CREB1	-0.04	-1.00	-0.96
KLHL13	-0.90	-1.39	-0.50
PES1	-0.31	-0.80	-0.48
MAX	-0.52	-0.65	-0.13
SEPT7	-0.25	-0.37	-0.12
NDRG3	-0.72	-0.35	0.36

PI3K/ AKT Signaling

Control

Symbol	Normalized
AR	11.59
IGF1	7.33
BCL2	3.10
PDPK1	1.08
AKT1	0.85
PTEN	0.57
CCND2	0.00
TIMP3	0.00
CCND1	-0.47
NFKB1	-0.93
GNRH1	-1.07
TP53	-1.52
VEGFA	-1.60
EGFR	-2.21
FOXO1	-6.92
TIMP2	-8.89
TNFRSF10D	-11.06
IL6	-11.32

miR-24-3p

Symbol	DU-145	MDA-PCa2b	logFC
IGF1	2.78	-3.07	-5.85
CCND2	2.58	0.89	-1.69
AR	0.72	-0.84	-1.55
BCL2	0.65	-0.86	-1.50
VEGFA	-0.13	-0.68	-0.55
EGFR	0.40	-0.03	-0.43
AKT1	-0.33	-0.70	-0.37
GNRH1	-0.49	-0.68	-0.19
PDPK1	-0.15	-0.23	-0.08
PTEN	-0.06	0.09	0.15
TIMP3	0.72	0.89	0.17
TP53	-0.59	-0.39	0.19
CCND1	-0.42	0.07	0.49
NFKB1	-0.67	0.03	0.70
TNFRSF10D	-0.25	0.89	1.14
TIMP2	-1.50	0.32	1.82
IL6	-1.34	0.75	2.09
FOXO1	-1.58	2.43	4.01

PTEN Signaling

Control

Symbol	Normalized
IGF1	7.33
PDPK1	1.08
AKT1	0.85
PTEN	0.57
TIMP3	0.00
GNRH1	-1.07
TP53	-1.52
VEGFA	-1.60
EGFR	-2.21
TIMP2	-8.89
IL6	-11.32

miR-24-3p

Symbol	DU-145	MDA-PCa2b	logFC
IGF1	2.78	-3.07	-5.85
VEGFA	-0.13	-0.68	-0.55
EGFR	0.40	-0.03	-0.43
AKT1	-0.33	-0.70	-0.37
GNRH1	-0.49	-0.68	-0.19
PDPK1	-0.15	-0.23	-0.08
PTEN	-0.06	0.09	0.15
TIMP3	0.72	0.89	0.17
TP53	-0.59	-0.39	0.19
TIMP2	-1.50	0.32	1.82
IL6	-1.34	0.75	2.09

Transcription Factor

Control

Symbol	Normalized
ETV1	13.80
AR	11.59
NKX3-1	4.46
RARB	4.20
EGR3	2.15
MSX1	1.98
SREBF1	1.39
MAX	0.44
CREB1	0.23
SUPT7L	0.17
SOX4	0.04
ERG	0.00
RBM39	-0.68
NFKB1	-0.93
DAXX	-1.27
TP53	-1.52
NRIP1	-1.78
ARNTL	-2.84
FOXO1	-6.92

miR-24-3p

Symbol	DU-145	MDA-PCa2b	logFC
AR	0.72	-0.84	-1.55
ETV1	0.72	-0.81	-1.53
NKX3-1	-0.27	-1.61	-1.35
NRIP1	-0.36	-1.37	-1.01
CREB1	-0.04	-1.00	-0.96
RARB	-0.85	-1.65	-0.79
ARNTL	-0.36	-1.00	-0.65
DAXX	-0.38	-0.78	-0.40
MAX	-0.52	-0.65	-0.13
MSX1	0.09	-0.03	-0.12
SREBF1	-1.66	-1.68	-0.02
SUPT7L	-0.18	-0.20	-0.02
RBM39	-0.35	-0.35	-0.01
ERG	0.72	0.89	0.17
TP53	-0.59	-0.39	0.19
SOX4	-0.39	-0.07	0.32
NFKB1	-0.67	0.03	0.70
EGR3	-0.80	0.54	1.34
FOXO1	-1.58	2.43	4.01

Upregulated in Prostate cancer

Control

Symbol	Normalized
KLK3	14.43
ETV1	13.80
IGFBP5	3.86
PDPK1	1.08
MTO1	0.76
SUPT7L	0.17
SOX4	0.04
RBM39	-0.68
DDX11	-1.34
ECT2	-1.62
CAMSAP1	-1.66
ARNTL	-2.84
HAL	-2.92
SOCS3	-7.69

miR-24-3p

Symbol	DU-145	MDA-PCa2b	logFC
ETV1	0.72	-0.81	-1.53
IGFBP5	0.72	-0.62	-1.33
ECT2	-0.29	-1.49	-1.20
KLK3	0.72	-0.33	-1.05
DDX11	-0.52	-1.17	-0.65
ARNTL	-0.36	-1.00	-0.65
MTO1	0.11	-0.30	-0.40
PDPK1	-0.15	-0.23	-0.08
CAMSAP1	-0.38	-0.46	-0.08
SUPT7L	-0.18	-0.20	-0.02
RBM39	-0.35	-0.35	-0.01
SOX4	-0.39	-0.07	0.32
SOCS3	-0.62	-0.16	0.46
HAL	0.03	0.85	0.82

Methylation Promoter

Control

Symbol	Normalized
AR	11.6
RARB	4.2
MSX1	2.0
APC	0.9
EDNRB	0.0
SLC5A8	0.0
RASSF1	-1.8
ZNF185	-2.8
CDKN2A	-5.6
CCNA1	-6.6
DKK3	-6.8
SFRP1	-7.0
GPX3	-7.1
TIMP2	-8.9
GSTP1	-10.1
DLC1	-10.5
TNFRSF10D	-11.1
CAV1	-13.6

miR-24-3p

Symbol	DU-145	MDA-PCa2b	logFC
AR	0.72	-0.84	-1.55
CCNA1	2.43	0.89	-1.54
APC	-0.07	-1.34	-1.27
GPX3	-0.43	-1.42	-0.99
RARB	-0.85	-1.65	-0.79
GSTP1	-0.20	-0.73	-0.53
MSX1	0.09	-0.03	-0.12
CDKN2A	-0.17	-0.27	-0.11
EDNRB	0.72	0.89	0.17
SLC5A8	0.72	0.89	0.17
ZNF185	1.14	1.38	0.24
RASSF1	-0.06	0.37	0.43
DLC1	0.41	0.89	0.48
DKK3	-0.16	0.89	1.06
TNFRSF10D	-0.25	0.89	1.14
CAV1	-0.73	0.89	1.63
TIMP2	-1.50	0.32	1.82
SFRP1	-1.05	0.89	1.95

Supplementary Figure 3: The fold-change analysis based on gene pathways.

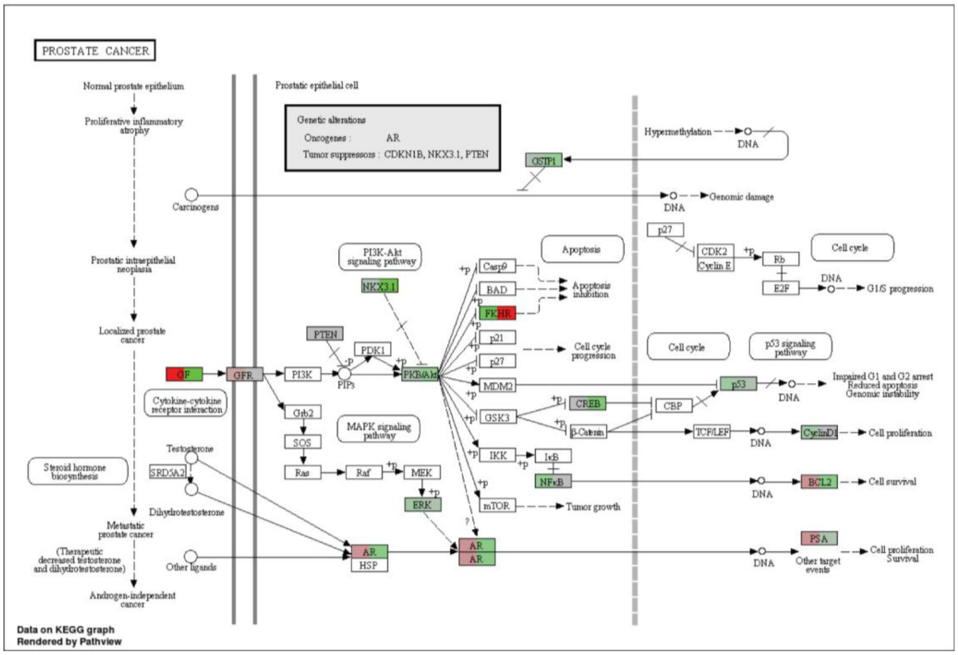

Supplementary Figure 4: Cancer-related gene pathway showing gene expression changes by miR-24-3p. Pathway map with representative genes altered by miR-24. "Pathview" analysis mapped qPCR array data to full map of "Pathway in Cancer" that has been defined by KEGG pathway database and renders pathway graph with the mapped data. The boxes surrounding gene symbols are illustrated by two colors. Left side color of the box shows expression change in DU-145 cells after miR-24 transfection and right side shows that for MDA-PCa-2b cells. The color indicates gene expression level in each cell line.

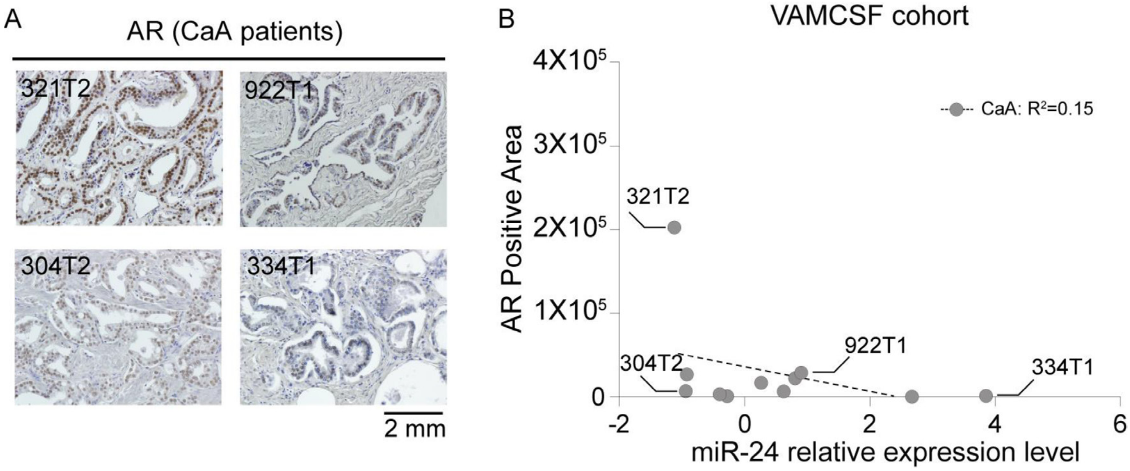

Supplementary Figure 5: Analyses of AR protein exp ression in CaA PCa tissues. (**A**) Immunohistochemical staining of AR protein expression. The staining is representative of 11 CaA (Grey-filled circles) PCa tissues. The number shown in the IHC image indicates the sample ID that is the same as shown in Figure 6B. (**B**) Association of AR expression positive area and miR-24 expression level in 11 CaA tissues.

Supplementary Table 1: Primer list of gene expression analysis

Gene name		Sequence	Tm
BcL2	Forward	GTGGATGACTGAGTACCTGAAC	58
	Reverse	GCCAGGAGAAATCAAACAGAGG	
ETV1	Forward	CTGAACCCTGTAACTCCTTTCC	58
	Reverse	AGACATCTGGCGTTGGTACATA	
IGF1	Forward	AGCAGTCTTCCAACCCAATTA	58
	Reverse	CAGAGCTGGTGAAGGTGAG	
IGFBP5	Forward	ACCTGAGATGAGACAGGAGTC	58
	Reverse	GTAGAATCCTTTGCGGTCACAA	
STK11	Forward	TGTCGGTGGGTATGGACAC	58
	Reverse	CCTTGCCGTAAGAGCCTTCC	
VEGFA	Forward	AGGGCAGAATCATCACGAAGT	58
	Reverse	AGGGTCTCGATTGGATGGCA	
AR	Forward	GACGACCAGATGGCTGTCATT	58
	Reverse	GGGCGAAGTAGAGCATCCT	
PDPK1	Forward	TTCCGAGCTGGAAACGAGTAT	58
	Reverse	GGTCTCTTGCCTTAGGGAAGAA	
B2M	Forward	CGTGTGAACCATGTGACTTTG	58
	Reverse	GGCATCTTCAAACCTCCATG	
PPP2R1B	Forward	CTTGTGTCAGTATTGCCCAGT	58
	Reverse	TGCTGCTTGTCGAAGTGTAGG	

Gene name		Sequence
AR-1	Forward	AGCTTTGTTTAAACTGCGTCCCAGAGCCTGGAGCCGTCTAGACTAG
	Reverse	CTAGTCTAGACGGCTCCAGGCTCTGGGACGCAGTTTAAACAAAGCT
AR-2	Forward	AGCTTTGTTTAAACTTGTTGCTGCCTCTTAGTCATCTAGACTAG
	Reverse	CTAGTCTAGATGACTAAGAGGCAGCAACAAGTTTAAACAAAGCT
IGFBP5-1	Forward	AGCTTTGTTTAAACTCCCCTGAGAAAAGACTGAGCCATCTAGACTAG
	Reverse	CTAGTCTAGATGGCTCAGTCTTTTCTCAGGGGAGTTTAAACAAAGCT
IGFBP5-2	Forward	AGCTTTGTTTAAACAAATGGGAGTTCTGGCTGAGCCCTCTAGACTAG
	Reverse	CTAGTCTAGAGGGCTCAGCCAGAACTCCCATTTGTTTAAACAAAGCT
IGF1	Forward	AGCTTTGTTTAAACCTGGGATTACAGGCATGAGCCACTCTAGACTAG
	Reverse	CTAGTCTAGAGTGGCTCATGCCTGTAATCCCAGGTTTAAACAAAGCT
VEGFA-1	Forward	AGCTTTGTTTAAACAACCCAGCCCTGGCGCTGAGCCTTCTAGACTAG
	Reverse	CTAGTCTAGAAGGCTCAGCGCCAGGGCTGGGTTGTTTAAACAAAGCT
VEGFA-2	Forward	AGCTTTGTTTAAACGACAAGGACGACTTGACTCGGTTCTAGACTAG
	Reverse	CTAGTCTAGAACCGAGTCAAGTCGTCCTTGTCGTTTAAACAAAGCT

Supplementary Table 2: 2 × 2 contingency table that describes the correlation between miR-24 expression level and race-related PCa

Data analyzed	CaA	AfA	Total
**High Expression**	5	1	6
**Low Expression**	46	80	126
**Total**	51	81	132

Cut off; Z = 2, Fisher's exact test,
$p = 0.0318$,
OR = 8.562693, 95% CI [0.9850 76.77].

Supplementary Table 3: Pathway analysis of the genes decreased after miR-24 transfection by CPDB

Pathway Name	Set Size	Candidates Contained	*p*-value	*q*-value	Pathway Source
Prostate cancer - Homo sapiens (human)	89	8 (9.0%)	5.87E-11	1.58E-08	KEGG
Pathways in cancer - Homo sapiens (human)	398	12 (3.0%)	1.67E-10	2.25E-08	KEGG
Integrated Pancreatic Cancer Pathway	170	9 (5.3%)	3.78E-10	3.39E-08	Wikipathways
Coregulation of Androgen receptor activity	61	5 (8.2%)	5.21E-07	3.51E-05	PID
AMPK signaling pathway - Homo sapiens (human)	124	6 (4.9%)	7.44E-07	4.00E-05	KEGG
FOXA1 transcription factor network	44	4 (9.1%)	5.28E-06	2.37E-04	PID
Regulation of Insulin-like Growth Factor (IGF) transport and uptake by Insulin-like Growth Factor Binding Proteins (IGFBPs)	21	3 (14.3%)	2.24E-05	8.00E-04	Reactome
Integrated Breast Cancer Pathway	64	4 (6.2%)	2.38E-05	8.00E-04	Wikipathways
PI3K-Akt signaling pathway - Homo sapiens (human)	347	7 (2.0%)	2.79E-05	8.15E-04	KEGG
AMPK Signaling	68	4 (5.9%)	3.03E-05	8.15E-04	Wikipathways
SIDS Susceptibility Pathways	159	5 (3.1%)	5.80E-05	1.36E-03	Wikipathways
Gastric Cancer Network 1	29	3 (10.3%)	6.05E-05	1.36E-03	Wikipathways
Apoptosis	84	4 (4.8%)	6.96E-05	1.44E-03	Wikipathways
Androgen receptor signaling pathway	89	4 (4.5%)	8.73E-05	1.68E-03	Wikipathways
Transcriptional misregulation in cancer - Homo sapiens (human)	179	5 (2.8%)	1.02E-04	1.74E-03	KEGG
CREB phosphorylation through the activation of CaMKK	6	2 (33.3%)	1.03E-04	1.74E-03	Reactome
MicroRNAs in cancer - Homo sapiens (human)	297	6 (2.0%)	1.15E-04	1.75E-03	KEGG
IGF signaling	36	3 (8.3%)	1.17E-04	1.75E-03	INOH
Focal Adhesion	190	5 (2.6%)	1.35E-04	1.91E-03	Wikipathways
HIF-1 signaling pathway - Homo sapiens (human)	103	4 (3.9%)	1.54E-04	2.07E-03	KEGG
